# Model Development on Adherence to Aspirin in Pregnancy: A Co‐Produced Qualitative Systematic Review and Meta‐Ethnography

**DOI:** 10.1111/hex.70703

**Published:** 2026-06-12

**Authors:** Raya Vinogradov, Eleanor Holden, Mehali Patel, Rowan Grigg, Linda Errington, Andy Husband, Judith Rankin, Vera Araujo‐Soares

**Affiliations:** ^1^ Faculty of Medical Sciences, Population Health Sciences Institute Newcastle University Newcastle upon Tyne Tyne and Wear UK; ^2^ NIHR Applied Research Collaboration North East and North Cumbria Newcastle upon Tyne Tyne and Wear UK; ^3^ Newcastle upon Tyne Hospitals NHS Foundation Trust Newcastle upon Tyne Tyne and Wear UK; ^4^ Non‐Affiliated Public Contributor Kenilworth Warwickshire UK; ^5^ Stillbirth and Neonatal Death Society (Sands) London Greater London UK; ^6^ Action on Pre‐eclampsia (APEC) Evesham Worcestershire UK; ^7^ School of Pharmacy Newcastle University Newcastle upon Tyne Tyne and Wear UK; ^8^ Medical Faculty Mannheim Heidelberg University Mannheim Baden‐Württemberg Germany

**Keywords:** adherence to medicine, aspirin, co‐production, meta‐ethnography, pre‐eclampsia prevention

## Abstract

**Objective:**

Low‐dose aspirin is proven to reduce the incidence of pre‐eclampsia, yet adherence remains low. This systematic review synthesises qualitative evidence on barriers and facilitators to low‐dose aspirin adherence in pregnancy and uses a co‐production approach to develop an explanatory model of this complex behaviour. The overall aim is to contribute to the development of a theory of adherence, targeting this health issue.

**Methods:**

*Data sources:* A search of electronic databases (Medline, Web of Science, CINAHL, PsycINFO, Embase, Scopus, Open Grey, Google Scholar, Prospero), charity and professional organisations’ archives was conducted using predefined terms. Citation searching was also performed. Searches were not time‐limited or language‐specific and completed in November 2024.

*Eligibility Criteria:* Studies, which included qualitative data on low‐dose aspirin determinants of adherence in pregnancy were included.

*Study Appraisal and Synthesis Methods:* Quality was assessed using the Critical Appraisal Skills Programme checklist. A meta‐ethnography approach with reciprocal translation and line‐of‐argument synthesis was used. The co‐production approach was utilised to streamline future intervention development by engaging key stakeholders in evidence synthesis, enabling them to translate evidence into action, support implementation and overcome the subjectivity inherent in meta‐ethnography. Co‐production activities followed the nominal group technique and structured discussions.

**Results:**

Out of 3757 items identified through systematic searches of published studies and grey literature, six studies were included in the review. No studies were excluded based on quality. Four 3rd‐order constructs with a total of 10 sub‐themes were identified: informational gap, verbal and non‐verbal health system communication, personal assets and autonomous control. In an explanatory model, we demonstrate that women are advised to take low‐dose aspirin in a context of lack of information and misconceptions (informational gap) with patchy and inconsistent messages from the health care system. Women ultimately control their decision about the use of low‐dose aspirin, however, the arrival at a decision depends on the utilisation of individual assets (unique personal or social characteristics inherited or acquired).

**Conclusions:**

Improving the quality and delivery of information for women and their support networks can reduce the strain on personal resources and make this essential preventive treatment more accessible and equitable.

**Patient or Public Contribution:**

A group consisting of representatives from two national charities and a service user worked alongside an academic team, contributing to all aspects of this work, including formulating the research question, participating in the selection of the search terms, conducting screening of the abstracts, data extraction, quality assessment, synthesis, drafting a graphic representation and this manuscript.

**Abbreviations**
ACTIVE: Authors and Consumers Together Impacting on eVidencE framework
APEC: Action on Pre‐eclampsia
CASP: Critical Appraisal Skills Programme
CINAHL: An index of English‐language and selected other‐language journal articles about nursing, allied health, biomedicine and healthcare
COM‐B: Capability, Opportunity, Motivation, and Behaviour model
EMBASE: A biomedical and pharmacological bibliographic database of published literature
EndNote X9: A reference management software used to store, organise and format bibliographic references and citations for academic writing
LDA: Low‐dose aspirin
PE: Pre‐eclampsia
PiCO: A framework used to formulate research questions (Population, Phenomenon of interest, context).
PRISMA: Preferred Reporting Items for Systematic Reviews and Meta‐Analyses
PROSPERO: The International Prospective Register of Systematic Reviews
Rayyan: A web‐based and desktop‐supported software tool designed to support systematic and scoping reviews, particularly during the study screening and selection phase
Sands: Stillbirth and Neonatal Death Society
Scopus: A large, multidisciplinary bibliographic database of peer‐reviewed literature
WoS: A multidisciplinary bibliographic database for peer‐reviewed literature and citation tracking

## 1 Introduction

Pre‐eclampsia (PE) is a pregnancy‐related syndrome affecting approximately 5% of pregnancies [1, 2] and the second most common direct cause of maternal death [3, 4], leading to approximately 60,000 deaths annually worldwide [5]. PE is also a major contributor to stillbirth, preterm delivery [6] and doubles short‐term healthcare costs for affected mothers and babies [7]. Additionally, PE increases long‐term cardiovascular risk in women affected by this syndrome during their pregnancies [8, 9, 10, 11]. Currently, delivery of the baby is the only treatment, underscoring the importance of early prediction and prevention [6].

Daily low‐dose (75 mg to 150 mg) aspirin (LDA) started before 16 weeks of pregnancy has been shown to reduce the incidence of PE amongst pregnant women at increased risk of the disease [12, 13, 14]. This preventative approach has been adopted globally [15, 16, 17]. Recent evidence indicates that adherence of 90% or higher is needed for optimal effect [18]. While adherence to aspirin during pregnancy in clinical trials is high (up to 96%) [19, 20], real‐world rates of adherence are alarmingly low, ranging from 6% to 54% [21, 22], diminishing the effectiveness of the preventative therapy. The World Health Organisation has identified non‐adherence as a public health challenge, particularly amongst younger individuals, women and those from low socioeconomic backgrounds [23, 24]. Addressing this issue is essential to maximise aspirin's preventive impact for those most in need, especially given the lack of alternative risk‐reduction strategies.

To address this issue, we have assembled a group of stakeholders, including service users, national charities, community practitioners, healthcare professionals and academics, to begin to develop an intervention. This manuscript represents an early stage of the intervention development process: evidence synthesis [25, 26], as a good understanding of the problem is essential for designing an effective solution. This work complements an earlier systematic review [27] that utilised the COM‐B framework [28] with elements of the medication adherence taxonomy [29] to guide the intervention development process. The COM‐B framework analysis identified key influences of the behaviour; ‘Insufficient knowledge’, ‘Necessity concerns balance’, ‘Access to medicine’, ‘Social influences’, and ‘Lack of Habit’ [27]. However, it did not allow an opportunity to utilise the rich expertise of public contributors, as it used a largely deductive approach and did not provide the co‐production group with a satisfactory contextual explanatory model. To build on the strength of the co‐production group with its variety of experiences, and to produce an explanatory model on aspirin adherence, a secondary synthesis of already identified literature was conducted using meta‐ethnography, with adaptations specific to the needs of co‐production.

## 2 Objective

This co‐produced review and meta‐ethnography aimed to increase understanding of the barriers and facilitators of adherence to LDA during pregnancy and to develop an explanatory model of this behaviour and its influencing factors.

## 3 Methods

### 3.1 Eligibility Criteria, Information Sources and Search Strategy

The modified PiCO (Population of interest, Context, Outcome) framework [30, 31] was used to frame the review question: ‘What are the barriers and facilitators of adherence to LDA during pregnancy?’. This review considered aspirin use for the prevention of pregnancy complications related to placenta‐mediated diseases.

Studies with a qualitative component, such as mixed‐methods research, interviews, surveys, focus groups and ethnographic studies, that reported direct quotes from participants were eligible for inclusion. While the database searches were conducted in English‐language databases, no language restrictions were applied during title and abstract screening. Searches were not time‐limited, covering publications from database inception until 18th November 2024. No restrictions were applied regarding study setting or country.

Search terms were co‐produced during a group meeting, ensuring they captured both lay language related to adherence and theoretical constructs explaining adherence behaviours. A predefined search strategy (detailed in Supporting Information) was used to conduct searches across multiple databases, including MEDLINE, PsycINFO, CINAHL, Web of Science (WoS), Scopus, EMBASE, Prospero and OpenGrey, to incorporate research reports, dissertations and conference proceedings. Additionally, websites and archives from key professional and charitable organisations, such as the Royal College of Obstetricians and Gynaecologists, Royal College of Midwives, Pre‐eclampsia Foundation, APEC and Sands, were searched, and relevant charities were contacted. Citation searches were performed for all included studies, and Google/Google Scholar was utilised to identify grey literature.

### 3.2 Study Selection

After deduplication in EndNote X9, all titles and abstracts were imported into Rayyan, a free web‐based screening tool, for blinded double screening by R.V., L.E., E.H. and M.P. based on predefined inclusion/exclusion criteria (see Supporting Information).

The review included studies with abstracts that focused on aspirin use for preventive purposes. Eligible studies were primary qualitative or mixed‐methods research exploring barriers and facilitators of aspirin use amongst pregnant women in any setting and of any age. Studies were excluded if they:

- Lacked a qualitative component,
- Focused on therapeutic aspirin use,
- Examined pre‐conceptional or postnatal preventative aspirin use,
- Were literature reviews (narrative or systematic), editorials, commentaries or educational materials.

Screening disagreements, primarily regarding study methodology, were minimal and resolved through a consensus meeting. All potentially relevant studies proceeded to full‐text review, which was conducted independently by two authors. Any ambiguous cases were discussed with a third reviewer (J.R.). Finally, citation tracking was conducted for all included studies.

### 3.3 Data Extraction

Data extraction was conducted independently by two reviewers (R.V. and E.H.). Key bibliographic, methodological and population‐related data were collected using a pre‐designed and piloted Excel worksheet. This review considered both first‐order constructs (direct participant quotes) and second‐order constructs (authors’ interpretations) for analysis. Data were extracted from results sections, tables, figures, and supporting materials and were made available to the review team in Word document format.

### 3.4 Risk of Bias

The Critical Appraisal Skills Programme (CASP) checklist for qualitative research [32] was used to assess the credibility, rigour and relevance of qualitative studies. It was chosen by the review team for its user‐friendly format. The quality of each included study was assessed independently by two blinded reviewers, and any discrepancies were resolved through a consensus meeting, where studies were reassessed and discussed. To further address uncertainties, reflective discussions were held with senior academic members (J.R. and V.A.S.). No studies were excluded based on the quality assessment.

### 3.5 Data Synthesis

#### 3.5.1 Meta‐Ethnography

Meta‐ethnography is a well‐established method for synthesising interpretive studies [33]. Through its seven‐step process, it enables a systematic and detailed exploration of how studies relate to one another while facilitating the development of higher‐order interpretations (theory generation or theoretical extensions) [34]. Although it originated from the field of education, this method of qualitative synthesis has been widely applied in a variety of health care research, inclusive of medication adherence [34, 35, 36, 37].

Given that meta‐ethnography is interpretive in its essence [33], allowing for the creation of third‐order constructs, it was deemed particularly suitable for a co‐production approach. This methodology provided an opportunity to integrate lived experiences alongside researcher interpretations, enriching the synthesis process [38].

To facilitate meaningful participation from lay contributors with no prior experience in meta‐ethnography, step‐by‐step training and detailed instruction were provided, and the seven‐step framework was adapted for online engagement with members of the public, as outlined in Table 1. Visualisation tools were used during videoconferencing sessions to help map the concepts and facilitate in‐depth discussion.

**Table 1:** Seven steps of meta‐ethnography and adaptation for co‐production.

Seven steps of meta‐ethnography	Adaptation for co‐production
Getting started	Building a co‐production team with breath of expertise
Deciding what is relevant	Discussion and developing searches using lay terms with additional training provision.
Reading the studies	Quality assessment, data extraction and reading (not modified) with additional training provision.
Determining how studies are related	a. Read the articles and think of what is common and what is different b. Record common/conflicting concepts/what stands out and submit
Translating studies into one another	Structured group discussion: Connecting/contrasting concepts (reflections)
Synthesising the translations	Structured group discussion: Reflecting on relations between the concepts in turn until a line of argument is established (common story)
Expressing the synthesis	Expressing results in a model (map and abstract)

Following familiarisation with the studies (step 3), a series of structured online meetings was conducted to support an analytic outcome reflecting a synthesis of perspectives rather than a unifocal interpretation. A shared understanding of relationships between the studies, as well as their translation and synthesis (steps 4–6), emerged through reflective listening to individual interpretations and the negotiated development of meanings and terms of reference that were later tested in further academic review with J.R. and V.A.S.

The development of third‐order constructs and the line of argument involved an iterative, reflexive process in which researchers and stakeholders collaboratively interrogated, translated and reconfigured interpretations across studies. This enabled engagement in an experience‐informed dialogic synthesis that surfaced latent meanings within the primary accounts (first‐ and second‐order constructs).

The final synthesis (step 7) was jointly articulated in both graphical and textual forms.

#### 3.5.2 Co‐Production

A co‐production approach was applied to the systematic review and meta‐synthesis to foster inclusive, collaborative and creative engagement, to produce a more nuanced theory that is relevant to a wide range of stakeholders, including service users [38], as well as to expedite future intervention development and its implementation [39].

Co‐production allows for a shift in power by moving from consultation to shared decision‐making and ownership [38] and thereby enhancing research impact. This can be achieved through the active involvement of key stakeholders throughout the entire research process, from knowledge generation to knowledge translation [40, 41]. Therefore, all efforts were made to conduct this review with the highest possible level of stakeholder involvement. Stakeholders contributed to formulating the research question, choosing methodologies, drafting the protocol, developing the search strategy, study selection, data extraction, risk of bias assessment, data analysis and synthesis, expression of the results, manuscript preparation and broader dissemination efforts, such as contributing to presenting the reviews’ findings. The extent of stakeholder engagement in this systematic review aligns with the ‘leading’ level of involvement as described in the Authors and Consumers Together Impacting on eVidencE (ACTIVE) framework, reflecting a shared leadership model in evidence synthesis [42].

The review team consisted of members from the public, charity, clinical and academic sectors, ensuring a diverse range of expertise and perspectives. This composition provided skills in systematic review methodology, meta‐ethnography, co‐production, clinical practice and lived experience. In particular, the team included representatives from two national charities, Sands (Stillbirth and Neonatal Death Society) and APEC (Action on Pre‐eclampsia), alongside a public contributor with experience of pre‐eclampsia and aspirin use in a subsequent pregnancy. Clinical expertise was represented by an obstetric sonographer, while methodological and academic expertise was provided by a medical librarian, a professor of maternal and child health, a professor of prevention specialising in complex intervention development and behaviour change, and a professor of pharmacy with expertise in meta‐ethnography and patient safety. The overarching aim of assembling this interdisciplinary team was to ensure that the findings of the review and synthesis could be effectively translated into intervention development and implementation in the future.

All members of the team were closely involved in all stages of the research process leading to this work, with two members (EH [lay] and VAS [academic]) having particularly close involvement in the framework analysis. Given the broader team's involvement and the interpretive, dialogic nature of the meta‐ethnographic approach, the influence of exposure to the COM‐B framework is considered to be limited.

The review was registered in the International Prospective Register of Systematic Reviews systematic review database (registration number CRD42022359718). Since the initial registration, the analytic approach shifted its focus from framework analysis based on the COM‐B model to an interpretive approach using meta‐ethnography.

## 4 Results

### 4.1 Study Selection

**Figure 1:**
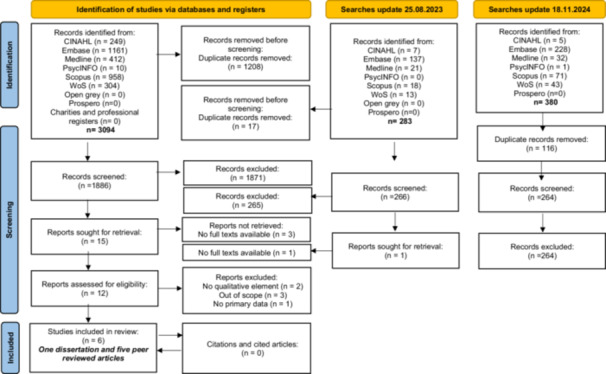
PRISAM flow diagram. *From:* Page MJ, McKenzie JE, Bossuyt PM, Boutron I, Hoffmann TC, Mulrow CD, et al. The PRISMA 2020 statement: an updated guideline for reporting systematic reviews. BMJ 2021;372:n71. doi: 10.1136/bmj.n7.

A total of 3757 titles were identified. Following the removal of duplicates (*n* = 1341), 2416 titles and abstracts were screened, and 16 records were sought for retrieval. Full texts for four records were not available, leaving 12 reports for full‐text eligibility screening. Subsequently, six reports were excluded: one due to a lack of primary qualitative data [43], three were out of scope for this review [44, 45, 46] and two had no qualitative element [47, 48]. Six studies met the inclusion criteria and were eligible for inclusion in this systematic review, as illustrated in Figure 1; one Master's degree dissertation [49], and five studies published in peer‐reviewed journals were included [50, 51, 52, 53, 54]. Citations for all included studies were back and forward searched and yielded no additional results.

In the process of study selection, a total of 68 discrepancies (2.8%) were resolved through a two‐step process: first, an initial meeting between the reviewers, followed by review by an expert (J.R. and V.A.S.) if consensus was not reached.

### 4.2 Study Characteristics

The included studies were published between 2019 and 2021, and originated from the US and Canada [52], the UK [49, 53, 54], the Netherlands [51] and Australia [50]. Four studies employed exclusively qualitative methodologies, one used a mixed‐methods approach [50], and another included an open‐ended question within a broader questionnaire study [52]. Various qualitative data collection methods were utilised, including responses to open‐ended questions within a questionnaire [52], interviews [49, 50, 53, 54] and focus group discussions [51].

Reflecting the diversity in data collection approaches, sample sizes ranged from 6 to 807 participants. All but one study focused on women at increased risk of PE, except Vestering et al. [51], who recruited participants from a low‐risk population. Five studies examined the use of LDA exclusively (with doses not always explicitly stated) [49, 50, 52, 53, 54], whereas Vestering et al. [51] investigated adherence to a polypill containing both low‐dose aspirin and calcium. Adherence levels among study participants varied from complete adherence to total non‐adherence. Two studies drew their analysis from the same data set; however addressed two separate research questions [53, 54]. A summary of the included studies is presented in Table 2.

**Table 2:** Methods and concepts.

Methods and concepts	Fenn et al. [49]	Shanmugalingam et al. [50]	Vestering et al. [51]	Ahmed et al. [52]	Vinogradov [53]	Vinogradov [54]
Year of publication	2019	2020	2019	2021	2021	2021
Methods	Qualitative	Mixed methods	Qualitative	Mixed methods	Qualitative	Qualitative
Data collection	Semi‐structured interviews	Interviews	Focus groups	Open‐ended question in questionnaire	Semi‐structured interviews	Semi‐structured interviews
Sample	13	6	25	807	14	14
Country	UK	Australia	Netherlands	US, Canada, UK and other countries	UK	UK
Population	At increased risk of pre‐eclampsia	At increased risk of pre‐eclampsia	Low risk	At increased risk of pre‐eclampsia	At increased risk of pre‐eclampsia	At increased risk of pre‐eclampsia
Medication	Low‐dose aspirin	Aspirin (100–150 mg)	Polypill (aspirin/calcium)	Low‐dose aspirin	Aspirin (75 mg)	Aspirin (75 mg)
Adherence level	71%‐ 100%	> 90%, < 90%	NA	NK	Complete non‐adherence, incomplete non‐adherence	Complete non‐adherence, incomplete non‐adherence
**Informational gap**						
Lack or little information	Lack of knowledge that aspirin is used to reduce the risk of pre‐eclampsia, how to take the medication, what formulation to take, when to take it or for how long.	/	Concerns about the lack of information about pre‐eclampsia. Safety is the most crucial condition for using a polypill, yet participants expressed doubts about the (long‐term) safety of aspirin.	Lack of information on the risks of low‐dose aspirin.	Participants demonstrated limited knowledge. Concerns were raised by participants about the (negative) effects of aspirin.	Inadequate knowledge. Women perceived their partners as needing more information.
Misconceptions	/	/	/	/	Misconceptions about the benefits of taking aspirin as preventative medication.	Not understanding preventative nature of aspirin.
**Health care system communication**						
Patchy information provision	Women were not always advised on exactly how to take the medication.	/	/	/	/	Knowledge was assumed due to her professional status and as a result information was not provided at the same level as to other high‐risk women.
Inconsistent messaging	/	The importance of consistent messaging and the negative impact of inconsistent messaging between health care professionals.	Information had to be unequivocal and was considered to be more reliable if provided by multiple sources.	/	Lack of coherence between hospital and community antenatal care and mixed messages from health care professionals.	/
Difficulties in access to medicine	Difficulties in replenishing aspirin.	/	/	/	Access to medication as a barrier.	/
**Personal assets**						
Personal identity (inclusive of beliefs and values, desire to enjoy pregnancy, perception of risk, personal and vicarious experience, trust)	Women are normalising risk. Women do own research, use information received in or following their previous pregnancy, or have access to information due to their occupation.	Trusting the Health Care System building on communications and relationships with the Health Care provider.	Trust (‘Medical professionals' expert opinion was considered the most trustworthy source of information’), yet participants had preconceptions about the use of medicine in pregnancy and rather avoided taking ‘medication’ in general and especially during pregnancy.	/	Lack of personal identification with the risk factors identified by health care professionals. Some women regarded themselves as someone who does not easily resort to taking medicines {…) unable to identify themselves as ‘medication takers’.	Lack of identity with someone who searches, avoidance of negative emotions and desire to adhere to initial intentions.
Skills (inclusive of practical skills and habits formed in the past, cognitive skills dictating the way women access and process information)	Strategies are needed to remember to take medicine.	Reminder strategies are important to overcome issues in relation to bedtime dosing of aspirin.	/	/	Lack of regular medication taking experience.	Lack of essential skills to actively search for information (not able to read). Women had difficulties in navigating the information ecosystem.
Physical and cognitive conditions inclusive of forgetfulness and tiredness	The main reasons for missing medication were linked to nausea and vomiting, as well as the taste of the medication.	Pill burden and a need in adherence to specific timing were associated with non‐intentional omission.	/	/	A very short interval between women discussing the option of aspirin prophylaxis and needing to decide about the use of aspirin. Poor memory and attention, a change in medical condition leading to a change in priorities.	Large amounts of new information delivered within a short time made it difficult for women to understand and process information in time to be able to ask questions. Information needs to be delivered in accessible format.
Social environment (how women navigate/use social environment, social comparisons, support)	Partners are instrumental in reminding to take medicine.	Positive impact of social media and good relationships with the Health Care Provider.	/	/	Unconditional support from relatives, irrespective of women's decision. Events such as a change in medical condition, significant social issues contribute to non‐adherence.	Active searching behaviours amongst this cohort were facilitated by social support.
**Autonomous control**						
Your body ‐your choice	Women have a choice regarding compliance to medication, and if they have been given the information, they are then able to make an informed decision regarding medication.	/	It was considered important that everyone, irrespective of one's risk of preeclampsia, should get the option to make this choice.	/	Relatives respect women's choice about the use of aspirin irrespective of the decision taken.	/

### 4.3 Risk of Bias

The most common methodological limitations were related to limited or inadequate reflection on the authors’ positionality and reflexivity, as well as insufficient rigour in qualitative data analysis, particularly in studies employing both qualitative and quantitative methods. The CASP checklist for the included studies is shown in Table 3.

**Table 3:** Quality assessment using the CASP tool for assessment of qualitative studies.

CASP category		Ahmed et al. [52]	Fenn et al. [49]	Shanmugalingam et al. [50]	Vestering et al. [51]	Vinogradov et al. [53]	Vinogradov et al. [54]
1	Was there a clear statement of the aims of the research?	Yes	Yes	Yes	Yes	Yes	Yes
2	Is a qualitative methodology appropriate?	Yes	Yes	Yes	Yes	Yes	Yes
3	Was the research design appropriate to address the aims of the research?	Yes	Yes	Yes	Yes	Yes	Yes
4	Was the recruitment strategy appropriate to the aims of the research?	Yes	Yes	Yes	Yes	Yes	Yes
5	Was the data collected in a way that addressed the research issue?	Yes	Yes	Yes	Yes	Yes	Can't tell
6	Has the relationship between the researcher and participants been adequately considered?	No	Yes	Can't tell	No	Yes	Yes
7	Have ethical issues been taken into consideration?	Yes	Yes	Yes	Yes	Yes	Yes
8	Was the data analysis sufficiently rigorous?	Can't tell	Yes	Can't tell	Yes	Yes	Yes
9	Is there a clear statement of findings?	Yes	Yes	Yes	Yes	Yes	Yes
10	How valuable is the research?	Yes	Yes	Yes	Yes	Yes	Yes

### 4.4 Synthesis of Results

Four key concepts emerged from the analysis: (1) Informational gap, (2) health care system communication, (3) personal assets and (4) autonomous control. The concepts identified across the studies were complementary rather than contradictory, enabling reciprocal translation. A detailed overview of these key concepts, along with their representation in individual studies, is provided in Table 2, and examples of the translation of first and second‐order constructs are available in Table 4. Each study contributed distinct meanings, with some conceptual overlap (Table 2). Both single and multiple concepts were given equal consideration during the interpretive process.

**Table 4:** Line of argument synthesis.

3rd order constructs	2nd order constructs	1st order construct examples
**Informational gap**: Lack or little information and misconceptions.	Lack or little knowledge amongst participants related to pre‐eclampsia, aspirin as preventative treatment, how to take aspirin. Little information provided about safety in the longer term.	‘I've heard of it … I never really … no …’ (ANA 2) ‘It's a blood thinner isn't it? I am assuming it keeps your heart pumping at the right level and your blood pressure down’. (ANA 4) [47] ‘I'm not sure I would [take aspirin] during pregnancy unless the data showed that it was safe’. (Term) [46] ‘I would want to know more about the side effects and other possible long‐term negative effects. That would make the difference for me, if they can say that it is proven to be safe and you don't have to worry’. (P02) [45]
**Signals from HC system**: Patchy information provision, inconsistent messaging and difficulties in access to medication.	Information is delivered not to all in need of information, information is inconsistent. Additional barriers to access medicine creates further inconsistency in messaging.	‘So, I found you don't get a lot of stuff explained to you because they automatically think you know… You don't get the same explanation as other people. you don't generally get information leaflets, you don't’. (ANA 14) [48] ‘It surprises me, I had never seen these figures and the severity of the condition. Considering that, I feel like our midwives haven't informed us properly’. (P21) [45] ‘I didn't realise that I had to carry on taking it because I only got like …there was no like repeat prescription…’ (ASPQUA09) [43] ‘The chemist told me that I should not take aspirin while I was pregnant despite my doctor's advice. This made my husband very concerned and discouraged me from taking the aspirin’. (Interview participant 1) [44]
**Personal assets**: Personal identity (inclusive of believes and values, desire to enjoy pregnancy, perception of risk, experience personal and vicarious, trust); Skills (inclusive of practical skills and habits formed in the past, cognitive skills dictating the way women access and process information); Social environment (how women navigate/use social environment, social comparisons, support); Physical and cognitive conditions inclusive of forgetfulness and tiredness	A mix of factors related to personal identity, skills, cognitive factors and social environment are used by women in a process of decision making as well as to establish medication taking routine.	‘I sort of knew that already before falling pregnant cos we'd already talked about it in the pre‐conception clinic anyway so I already knew …’ (ASPQUA03) [43] ‘Speaking to other women that have been through it (preeclampsia) and that are going through it–you know finding friends who are on or who have taken aspirin in pregnancy, who are going through similar things gave me comfort in taking it’. (Interview participant 6) [44] ‘I'd rather not take anything chemical, but if it is proven to be good, I would consider it’. (P21) [45] ‘I lost my sister when I was 8 weeks pregnant and that was a very strange one because none of my family knew, so dealing with that was a bit intense. I think the worst things that could happen, we also had to move out of our flat … ‘(ANA 13) [47] ‘A lot of people aren't fantastic readers either. To some people it's just a lot of scribble on paper’. (ANA 2)[48]
**Autonomous control** (‘Your body ‐your choice’)	Women should have a choice about the use of aspirin, and this choice is likely to be supported.	‘People have to take their own responsibility. You know it's their own bodies, it's their own pregnancies, they know the risks, they know the benefits, you can spoon feed people as much as you like but if they're gonna take it they're gonna take it’. (ASPQUA01) [43] ‘Not giving people a choice is worse than the possibility of worrying them by telling. If you worry about it and there is something available, then at least you can do something about. ‘ (P11) [45] ‘And he said [partner] only you can make the choice’ (ANA 11) [47]

### 4.5 Informational Gap

The Informational gap concept was comprised of two subthemes: (a) Little or lack of information and (b) misconceptions.

The subtheme ‘Little or lack of information’ was represented in five out of six studies, covering topics such as the disease itself, the medication, its correct usage [49, 51, 53, 54] and the lack of information regarding the negative consequences of medication use [51, 52, 53]. A subtheme, ‘Misconceptions’ was present in two studies [53, 54] and was related to a poor understanding of the preventative nature of aspirin treatment.

You only get a bare minimum [information]. *[* *54* *]*

‘I'm not sure I would [take aspirin] during pregnancy unless the data showed that it was safe’. [52]

‘It's a blood thinner, isn't it? I am assuming it keeps your heart pumping at the right level and your blood pressure down’. [53]

### 4.6 Health Care System Communication

The signals from the Health Care System concept covered explicit and implicit communications with subthemes: (a) Patchy information provision, (b) inconsistent messaging and (c) difficulties in access to medicine. Information was not always provided [49] and, in some cases, knowledge was assumed [54]. Health care providers were sending inconsistent messages that changed from setting to setting [49, 50], from health care professional to health care professional [53], while women were stressing the importance of consistent and unequivocal messaging [51]. Additionally, difficulties in access to medicine created further inconsistencies in messaging. Women and pregnant people faced additional barriers to obtaining aspirin, which compounded the existing inconsistencies in the health care system's communication [49, 53].

‘The midwives didn't want to take any responsibility over that. They are quite happy to push vitamins folic acid (but not aspirin)’. [53]

‘I found you don't get a lot of stuff explained to you because they automatically think you know. You don't get the same explanation as other people’. [54]

‘The chemist told me that I should not take aspirin while I was pregnant despite my doctor's advice’. [50]

### 4.7 Personal Assets

The ‘Personal assets’ concept played a role in supporting and hindering decision‐making and medication‐taking processes. This concept represented inherited and acquired personal characteristics under four subthemes: (a) Personal identity (inclusive of beliefs and values, desire to enjoy pregnancy, perception of risk, experience personal and vicarious, trust) [49, 50, 51, 53, 54]; (b) skills (inclusive of practical skills and habits formed in the past, cognitive skills dictating the way women access and process information) [49, 50, 53, 54]; (c) physical and cognitive conditions, inclusive of forgetfulness and tiredness [49, 50, 53, 54]; (d) social environment (the way women navigate and use social environment, social comparisons, support) [49, 50, 53, 54].

No, it was fine I can take it I just, I don't like my body relying on medicine. Like if I've got a headache, I don't like taking paracetamol I'd rather my body gets rid of it on its own *[* *53* *]*

‘I sort of knew that already before falling pregnant cos we'd already talked about it in the pre‐conception clinic anyway’. [49]

‘I had a good rapport with my doctor early on, which helped me trust the healthcare system’ [50]

‘Speaking to other women that have been through it (preeclampsia) and that are going through it–you know finding friends who are on or who have taken aspirin in pregnancy, who are going through similar things gave me comfort in taking it’. [50]

‘A lot of people aren't fantastic readers either. To some people it's just a lot of scribble on paper’. [54]

### 4.8 Autonomous Control

The last concept was ‘Autonomous control’, reflected in three studies outlining that women have a choice and can decide on the use of aspirin if they have been provided with the information [49], that they should have a choice [51], and their choice of action is respected [53].

‘People have to take their own responsibility. You know it's their own bodies, it's their own pregnancies, they know the risks, they know the benefits, you can spoon feed people as much as you like but if they're gonna take it they're gonna take it’. [49]

‘Not giving people, a choice is worse than the possibility of worrying them by telling. If you worry about it and there is something available, then at least you can do something about’. [51]

‘And he said only you can make the choice’. [53]

### 4.9 Line of Argument and Conceptual Model

**Figure 2:**
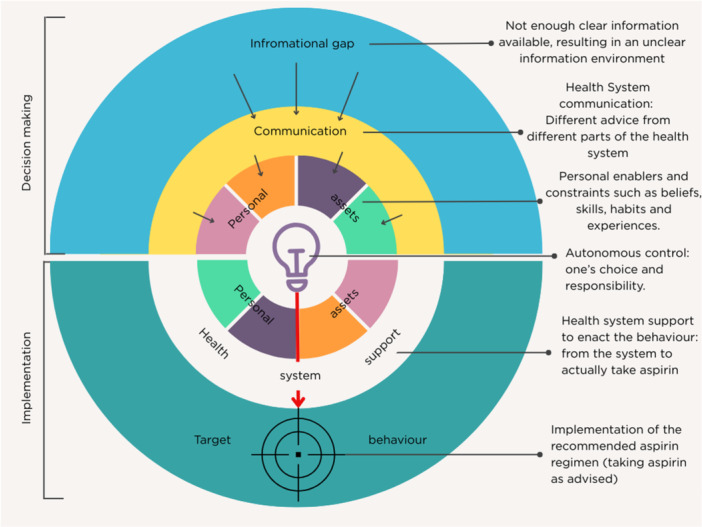
Conceptual model of aspirin‐taking behaviour during pregnancy.

The line of argument was co‐produced during a collaborative meeting, where members of the co‐production team took turns comparing concepts, considering all included studies. Through several iterative discussion cycles, the team explored the relationships among third‐order constructs and gradually reached a shared interpretation. Agreed results are represented schematically in a conceptual model (see Figure 2).

Pre‐eclampsia prevention is offered within a context of limited information and misconceptions (informational gap, the upper outer semicircle), largely due to lack of awareness of pre‐eclampsia and the overall scarcity of information on aspirin use in pregnancy in the public domain.

Within the context of the informational gap, the healthcare system delivers inconsistent messages, which vary between settings (community vs. hospital) and among healthcare professionals (yellow semicircle below the informational gap). A key inconsistency relates to access to aspirin, leading to further confusion and indecision. As a result, women are left to interpret these conflicting signals on their own and finally make a decision.

Women have autonomous control and responsibility (agency) over the decision‐making process (the ‘bulb’ core of the model). However, the decision‐making as well as medication‐taking processes (green lower outer circle) rely heavily on personal assets, including inherited or acquired characteristics such as beliefs, values, the desire to enjoy pregnancy, perception of risk, past experiences, trust, practical and cognitive skills, habits, ability to navigate social environments and physical and cognitive conditions (located above the agency/decision making core with different coloured slices).

While the responsibility for decision‐making is placed on women by themselves, their relatives and the healthcare system, they are not provided with all the necessary tools to make informed choices.

Below the core, there are personal assets again, as those are used to translate decisions into action, with an empty space below (in white) signifying no evidence of institutional support to the enactment of the behaviour (aspirin intake).

## 5 Comment

### 5.1 Principal Findings

This systematic review involving a meta‐ethnography approach identified four key concepts outlining the use of aspirin in pregnancy: Informational gap, communication signals from the health care system, personal assets and autonomous control. In a context of informational gap, related to the use of aspirin in pregnancy, women and pregnant people strive to achieve control while heavily relying on their personal assets to interpret the often‐conflicting signals from the healthcare system.

### 5.2 Comparison With Existing Literature

The lack of knowledge related to the use of medicines in pregnancy is reflective of a history of exclusion of women, let alone pregnant women, from pharmaceutical research due to safety concerns. Using the example of aspirin, where there is good evidence of effectiveness in the prevention of early onset of PE and decades of experience of aspirin use in clinical settings, dose optimisations studies of aspirin for PE prevention have just started to emerge. The emerging scientific knowledge related to the negative effects of aspirin is relatively new and is yet unlikely to be mobilised soon by a wide range of healthcare professionals supporting women and pregnant people throughout their journey.

Lay knowledge about pre‐eclampsia and preventative strategies is well studied and globally reported to be low [55, 56, 57, 58, 59, 60]. Gaps in knowledge were also identified within health professional settings [61]. Despite the presence of clear guidelines, opportunities to provide advice on aspirin use are missed at times [62, 63] or in places where knowledge is evident, resources to facilitate knowledge sharing are lacking [64]. This is worse in middle and low‐income countries, as identified in a recent systematic review [65]. This is further impacted by attitudes towards aspirin from the various health care professionals women encounter when attempting to obtain aspirin. Driven by patients’ safety, pharmacists and general practitioners may feel an urge to reassess the need and safety of aspirin use in pregnancy [47, 66], with specialised obstetricians being a more likely group to advise on aspirin use for women at risk of PE compared to any other health professional group [67]. Knowing that women often need to speak to several healthcare professionals during pregnancy, such as midwives, obstetricians, radiographers, pharmacists and general practitioners, we acknowledge that these professionals may hold different views on the use of aspirin in pregnancy and perceive their role differently. It is also known that some HCPs opinions carry more weight than others. For example, women may see advice from a GP or pharmacist as more authoritative than advice from a midwife or radiographer. This situation creates barriers to adherence, meaning in some cases, women have to navigate contradictory advice. In other cases, women are unable to access aspirin at all [53]. All of these factors contribute to overall non‐adherence rates [47].

When facing conflicting signals in a context of little knowledge, women use their personal assets or resources to navigate the decision‐making processes and to establish medication‐taking routines. Women of a lower socioeconomic background [68], smokers [18], those from ethnic minority groups [69] and of a younger age [18], are less likely to adhere to aspirin during pregnancy. Further, Lin et al. suggest that action and coping planning, as well as relationships with a partner, have a positive association with aspirin adherence amongst pregnant women [70]. Those are reflective of adherence‐associated factors reported in an updated systematic review of systematic reviews in the non‐pregnant population; with factors such as socioeconomic status and social support positively associated with adherence, while factors such as costs for the patient, and being part of ethnic minorities, are negatively associated with adherence [71]. Thus, factors indicative of the presence of high‐level personal or social assets are associated with higher levels of adherence, while low‐level assets are associated with non‐adherence.

Our argument that women take ownership of their decisions is supported by findings from Olson et al., who reported that women who decided not to take aspirin, that is, demonstrating intentional non‐adherence, were unlikely to consult their medical providers on the matter again [47].

In a context of clinical research, new interventions have emerged trying to address the informational gap amongst women [72]; however, addressing the information gap as a single issue and only with women is unlikely to be sufficient.

Social cognitive theory [73] aligns well with the explanatory model we describe. In our systematic review, we found evidence suggesting that women's agency and the enactment of behaviours, in this case medication‐taking behaviour, depend heavily on personal assets (capital or resources) that inform individual decision‐making and enactment. Social cognitive theory conceptualises these influences as determinants of human behaviour, incorporating behavioural factors such as skills, practice and self‐efficacy; cognitive factors such as knowledge, attitudes and expectations; and environmental factors such as social norms. These determinants vary considerably between individuals, indicating that future interventions will need to be tailored to achieve effectiveness. Volitional constructs might also need to be addressed, such as action planning, coping planning and habit formation [74].

Compared with the COM‐B approach used deductively in a previous systematic review [27], which helped identify behavioural influences to be targeted by a future intervention to develop, the co‐produced meta‐ethnography provided a deeper conceptual understanding of behaviour within a complex system, through the lens of service users. This approach highlighted the critical role of the health care system and shifted the emphasis for change from individuals to systemic responsibility, highlighting that the system needs to accommodate the needs of the people who hold different assets (identities, abilities, values and skills).

### 5.3 Co‐Production Reflection by Members

Service users and representatives from two national charities (APEC and Sands) worked collaboratively on this review alongside clinical professionals and academics. Together, we co‐produced and shared evidence to support the better use of aspirin in pregnancy for the prevention of pre‐eclampsia. We were united by our shared commitment to reducing health inequalities, enhancing safety in perinatal services and improving pregnancy outcomes.

By contributing directly to the review, public contributors gained valuable insight into the research process. As they engaged with the literature and explored how it could be integrated into a coherent model, they amplified not only their own voices and experiences, but also those of the many families supported by the charities. In doing so, the team aimed to ensure that the research more accurately reflected the realities of lived experience.

The group dynamic felt collaborative and respectful, with everyone's contributions valued and a strong collective commitment to the review's success.

### 5.4 Strengths and Limitations

The main strength of this work is the utilisation of a co‐production approach to streamline knowledge mobilisation amongst key stakeholders and future intervention development. This was achieved by engaging service users, two national charities, community and health care practitioners, as well as academics in evidence synthesis, enabling them to translate evidence into action and to support implementation in later stages of the project. Our group finds the use of meta‐ethnography, an analytical approach, well‐suited for use in co‐production, allowing equal contribution by lay team members. The interpretative and transparent nature of meta‐ethnography allowed a deeper level of synthesis that amplified the voices of the service users in the evidence synthesis work. It allowed us to develop an explanatory framework highlighting issues of inequalities in a process of decision‐making in a context of aspirin use in pregnancy that is likely applicable beyond this context.

Yet, with the benefits of the interpretive nature of meta‐ethnography come its limitations of subjectivity of interpretation. Our group hopes that we were able to overcome this limitation by including perspectives from a diverse group of people in the analytical process.

We approached this work with a comprehensive search strategy developed with and conducted by an experienced medical librarian, with searches not being limited to abstracts in English and all titles and abstracts being screened by two blinded members of the review team. Despite comprehensive searches, we acknowledge that some grey literature may have been missed, as it is not always accessible through public search platforms.

It is important to acknowledge that although the amount of data contributed by each study varied and some studies reported overlapping data, each addressed a unique aspect related to adherence to LDA during pregnancy. Included studies complemented one another and collectively contributed to the development of the explanatory model of aspirin‐taking behaviour.

Lastly, this area of research is not particularly evidence‐rich. We were unable to identify published or unpublished qualitative literature prior to 2019, and all included studies originated from high‐income countries (USA, Canada, UK, the Netherlands and Australia). This likely reflects the broader implementation of aspirin use for pre‐eclampsia prevention over the past two decades in high‐income settings, alongside limited availability of recommendations, late initiation of antenatal care and access to medication in low‐ and middle‐income countries [75]. Therefore, the interpretation and application of the study findings are limited to the literature examined, which was produced in high‐income settings. Further research is warranted to examine barriers and facilitators to the implementation of aspirin prophylaxis in low‐ and middle‐income settings, as this may provide valuable insights into behaviour and inform the development of contextually appropriate interventions for healthcare systems and consumer practices in low‐ and middle‐income countries.

### 5.5 Conclusions and Implications

Understanding and developing a conceptual model of adherence to aspirin in pregnancy increases the opportunities to support women and their networks by improving the quality and delivery of information, removing barriers to medication access and providing strategies to support medication‐taking routines. Enhancing these aspects can help reduce the burden on personal resources and, in turn, mitigate inequalities in pre‐eclampsia prevention.

Results of this study will directly feed into an intervention development process to support women and pregnant people at increased risk of pre‐eclampsia in their decision‐making and adherence to aspirin.

## Author Contributions

**Raya Vinogradov:** conceptualisation (lead), methodology (equal), resources (support), data curation (lead), formal analysis, software (support), writing – original draft (lead), visualisation (lead), writing – review and editing (lead), project administration (lead), investigation (lead), funding acquisition (lead), validation (lead). **Eleanor Holden:** conceptualisation (supporting), writing – review and editing (supporting), visualisation (supporting), methodology (supporting), formal analysis (supporting), data curation (supporting), validation (supporting), investigation (equal). **Mehali Patel:** writing – original draft (supporting), conceptualisation (supporting), investigation (equal), methodology (supporting), validation (supporting), visualisation (supporting), writing – review and editing (supporting), formal analysis (supporting), data curation (supporting). **Rowan Grigg:** conceptualisation (supporting), investigation (equal), writing – review and editing (supporting), visualisation (supporting), validation (supporting), methodology (supporting), formal analysis (supporting), data curation (supporting). **Linda Errington:** supervision (supporting), investigation (equal), writing – original draft (supporting), writing – review and editing (supporting), methodology (supporting). **Andy Husband:** conceptualisation (supporting), methodology (supporting), validation (supporting), writing – review and editing (equal), supervision (supporting). **Judith Rankin:** supervision (equal), data curation (supporting), validation (supporting), methodology (supporting), conceptualisation (supporting), writing – review and editing (equal), investigation (supporting). **Vera Araujo‐Soares:** conceptualisation (supporting), investigation (supporting), writing – original draft (supporting), writing – review and editing (equal), validation (supporting), methodology (supporting), supervision (equal), visualisation (supporting).

## Ethics Statement

Ethical approval was not required for this systematic review, as the study involved the analysis of previously published data and did not include the collection of primary data from human participants.

## Conflicts of Interest

The authors declare no conflicts of interest.

## Supporting information

Supporting File 1

Supporting File 2

Supporting File 3

Supporting File 4

## Data Availability

Data retrieved from publicly available resources: Five are published in peer‐reviewed journals, and one dissertation is available from Newcastle University.
