# Supplementary material for: Model Development on Adherence to Aspirin in Pregnancy: A Co‐Produced Qualitative Systematic Review and Meta‐Ethnography
Source: Health Expect. 2026 Jun 12;29(3):e70703. doi: 10.1111/hex.70703 (PMC13261684; doi:10.1111/hex.70703)
Supplement: Supplementary file 2 — Supporting File 2 [file HEX-29-e70703-s002.docx]

# Inclusion/exclusion criteria

**Screening guide**

**Barriers and facilitators of adherence to low-dose aspirin during pregnancy:**

**A co-produced systematic review and COM-B framework synthesis of qualitative evidence**

**Background:**

The aim of this review is to identify and synthesise qualitative evidence on barriers and facilitators (experiences) of use of low-dose aspirin in pregnancy.

1. **Inclusion and Exclusion criteria:**

| **Inclusion** | **Exclusion** |
| --- | --- |
| Use of **aspirin for preventative** reasons | Aspirin used to treat health condition |
| Research describing **experiences i.e. research using** qualitative methods | Studies without qualitative component |
| Primary studies (studies collecting new data) | Literature reviews such as narrative and systematic reviews; editorials or commentaries |
| Experiences of women/pregnant people | Use of aspirin before or after pregnancy |
| Any country, any setting i.e, it could be within primary or secondary care, public/private services. | Aspirin use in other human populations or animals |
| Any age |  |
| Any language as long as abstract is available in English |  |

**Notes**

- Aspirin could be used at any dose (60-300 mg)
- Aspirin could be advised or prescribed
- Qualitative studies tend to describe experiences of groups of people, it could be observations, interviews, focus groups, analysis of text, or open question in questionnaires.
- When and if you come across the study with a different population (let’s say pharmacists) but they talk about women’s experience of using aspirin please enter ‘maybe’ and we can discuss later.
- Please add any queries you have in WhatsApp as all of us have access to it and can view responses. Remember if this is not clear to you it’s very likely that this will be unclear to other members of the team too.
